# Soft‐Templated Electroless Synthesis of Mesoporous Metal Films on Non‐Conductive Substrates

**DOI:** 10.1002/smll.202505676

**Published:** 2025-08-19

**Authors:** Mandy H. M. Leung, Hirokatsu Miyata, Tokihiko Yokoshima, Yusuke Asakura, Minsu Han, Daigo Natsuhara, Miharu Eguchi, Chia‐Hung Liu, Yusuke Yamauchi

**Affiliations:** ^1^ Department of Materials Process Engineering Graduate School of Engineering Nagoya University Nagoya Aichi 464‐8603 Japan; ^2^ School of Advanced Science and Engineering Waseda University Shinjuku‐ku Tokyo 169‐8555 Japan; ^3^ Research Center of Urology and Kidney and Department of Urology School of Medicine College of Medicine Taipei Medical University 250 Wu‐Hsing Street Taipei 11031 Taiwan; ^4^ Department of Urology Shuang Ho Hospital Taipei Medical University 291 Zhongzheng Road, Zhonghe District New Taipei City 23561 Taiwan; ^5^ Australian Institute for Bioengineering and Nanotechnology (AIBN) The University of Queensland Brisbane QLD 4072 Australia

**Keywords:** electroless deposition, mesoporous platinum films, microcontact printing

## Abstract

Here, a novel electroless deposition approach is proposed that utilizes the spontaneous assembly of micelles and metal ions on non‐conductive substrate surfaces, induced by reducing agents, to fabricate mesoporous Pt (mPt) films. By utilizing the effective interaction between deposited initial nuclei and 3‐aminopropyltriethoxysilane (APTES), the electroless deposition of mPt is achieved on APTES‐modified glass surfaces during chemical reduction. Using this approach, mPt film on 4‐inch glass wafers is successfully prepared. Additionally, the potential of this method is explored for fabricating mPt patterns using microcontact printing of APTES. The results demonstrate the successful patterning of 15 µm‐wide mPt stripes on glass substrate. This soft‐templated electroless approach overcomes the limitations of current electrochemical assembly methods regarding substrate conductivity, shape, and size. Moreover, this electroless reduction strategy is expected to be compatible with various substrates, opening new avenues for researchers to explore applications of mesoporous metal films.

## Graphical abstract

Electroless deposition of mesoporous Pt (mPt) films on non‐conductive substrates by chemical reduction and utilizes the spontaneous assembly of micelles and metal ions. The substrate surface functionalized with APTES facilitates the assembly of micelles and subsequent metal nucleation. Using this approach, large mPt films on 4‐inch glass wafers is successfully fabricated. Furthermore, well‐defined 15 µm‐wide stripes of mPt by microcontact printing of APTES is patterned.

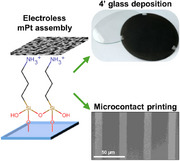

## 1 Introduction

Mesoporous materials with their high surface area and large pore volume are indispensable in diverse applications such as gas storage and separation, catalysis, catalyst supports, and adsorption.^[^ ^1^, ^2^ ^]^ Traditionally, these materials have been synthesized through sol‐gel reactions involving metal alkoxides (or metal salts), carbonization of organic polymers, or coordination reactions between metal ions and organic ligands. These approaches, however, have predominantly yielded compositions limited to metal oxides (e.g., silica, titania, alumina),^[^ ^3^ ^]^ carbon,^[^ ^4^, ^5^ ^]^ and metal‐organic frameworks.^[^ ^6^ ^]^ Recently, increasing attention has been directed toward controlling electron mobility within mesoporous frameworks. Leveraging this capability, mesoporous materials have been explored in photodetectors, plasmonic substrates, electrochemical sensors (including biosensors), battery electrodes, and electrocatalysts fields where efficient electron movement within the frameworks is crucial. Such properties are beyond the reach of traditional mesoporous materials and efforts have therefore focused on designing materials with conductive or semiconducting frameworks, particularly those featuring zero or narrow band gaps.^[^ ^7^ ^]^

Historically, achieving such frameworks often relied on lyotropic liquid crystals (LLCs) formed by highly concentrated surfactants or amphiphilic molecules as templates.^[^ ^8^, ^9^ ^]^ While effective in early studies, the high viscosity of LLCs posed significant challenges in handling and restricted their applicability to a limited range of compositions. In contrast, the electrochemical assembly of block copolymer‐based micelles in aqueous solutions presents a versatile and efficient strategy for synthesizing mesoporous materials with diverse compositions.^[^ ^10^ ^]^ This strategy extends beyond single metals to include binary alloys, multi‐component high‐entropy alloys, and even chalcogenides, demonstrating its potential for a wide array of advanced applications. The electrochemical methods offer two primary approaches: chemical reduction using reducing agents to synthesize mesoporous nanoparticles;^[^ ^11^, ^12^, ^13^ ^]^ and electrochemical deposition using external power sources to produce mesoporous films.^[^ ^14^, ^15^ ^]^ Mesoporous nanoparticles can be hybridized with conductive matrices to create desired forms that are ready for practical applications. On the other hand, mesoporous films show remarkable versatility. By adjusting deposition time and applied potentials, it is possible to precisely control film thickness and pore arrangement on a wide variety of conductive substrates. Notably, patterned mesoporous metal films present significant advantages for enhancing device performance in applications such as sensors, electronic devices, and optical systems.

A variety of patterning techniques have been developed for mesoporous materials, with photo‐ or electron‐lithography being the most widely utilized.^[^ ^16^, ^17^, ^18^, ^19^, ^20^ ^]^ Traditionally, precursor solutions containing surfactants and metal alkoxides are introduced into confined areas defined by lithography techniques. Once the sol‐gel reactions followed by the lift‐off process are completed, diverse patterns can be fabricated. However, these methods are typically limited to non‐metallic materials. Recently, our group demonstrated the successful electrochemical deposition of mesoporous metal films on patterned conductive surfaces prepared using photolithography.^[^ ^21^ ^]^ While this approach represents significant progress, the inherent limitation remains: electrochemical deposition only occurs on conductive surfaces. This restriction poses challenges for advancing mesoporous metal applications. To overcome this limitation, it is highly desirable to develop methods that enable the deposition of mesoporous metal films on non‐conductive surfaces while allowing pattern control through conventional and accessible techniques, such as microcontact printing (µCP). However, existing synthetic processes have yet to accomplish this goal.

Here, we propose a novel electroless deposition approach that utilizes the spontaneous assembly of micelles and metal ions on non‐conductive substrate surfaces, induced by reducing agents, to fabricate mesoporous metal films. An important aspect of this approach is the modification of glass surface with 3‐aminopropyltriethoxysilane (APTES), a technique that has been widely used to immobilize proteins and nanoparticles onto the surface of glass or silicon substrates.^[^ ^22^, ^23^, ^24^, ^25^ ^]^ While polydopamine is also commonly employed for metal adhesion due to its universal coating ability and strong binding properties,^[^ ^26^, ^27^, ^28^ ^]^ we selected APTES for its ability to form a stable, covalently bonded monolayer on hydroxylated surfaces. Its thin, electrochemically inert layer minimizes impedance and background signals, making it better suited for applications requiring efficient electron transfer. Furthermore, metalate ions and metallic seed particles during chemical reduction are capable of binding to the APTES‐modified surface.^[^ ^24^ ^]^ With this electroless approach, we successfully prepare a mesoporous Pt (mPt) film on a 4‐inch glass wafer. We further explore the potential of this electroless approach for fabricating mPt patterns with µCP of APTES, and our results show that 15 µm wide stripes of mPt are patterned over the entire area of glass substrates. Our electroless approach not only eliminates the limitations on substrates imposed by current electrochemical assembly methods, but also enables the fabrication of self‐standing mPt films and micropatterns. While surface hydroxylation (e.g., via UV/ozone treatment) is required for APTES modification, this electroless method can still be applied to a variety of substrates, including certain hydrophobic materials. As such, it has potential to broaden the use of mesoporous metal films in applications beyond electrochemical sensing and electrocatalysis.

## 2 Results and Discussion

**Figure 1:**
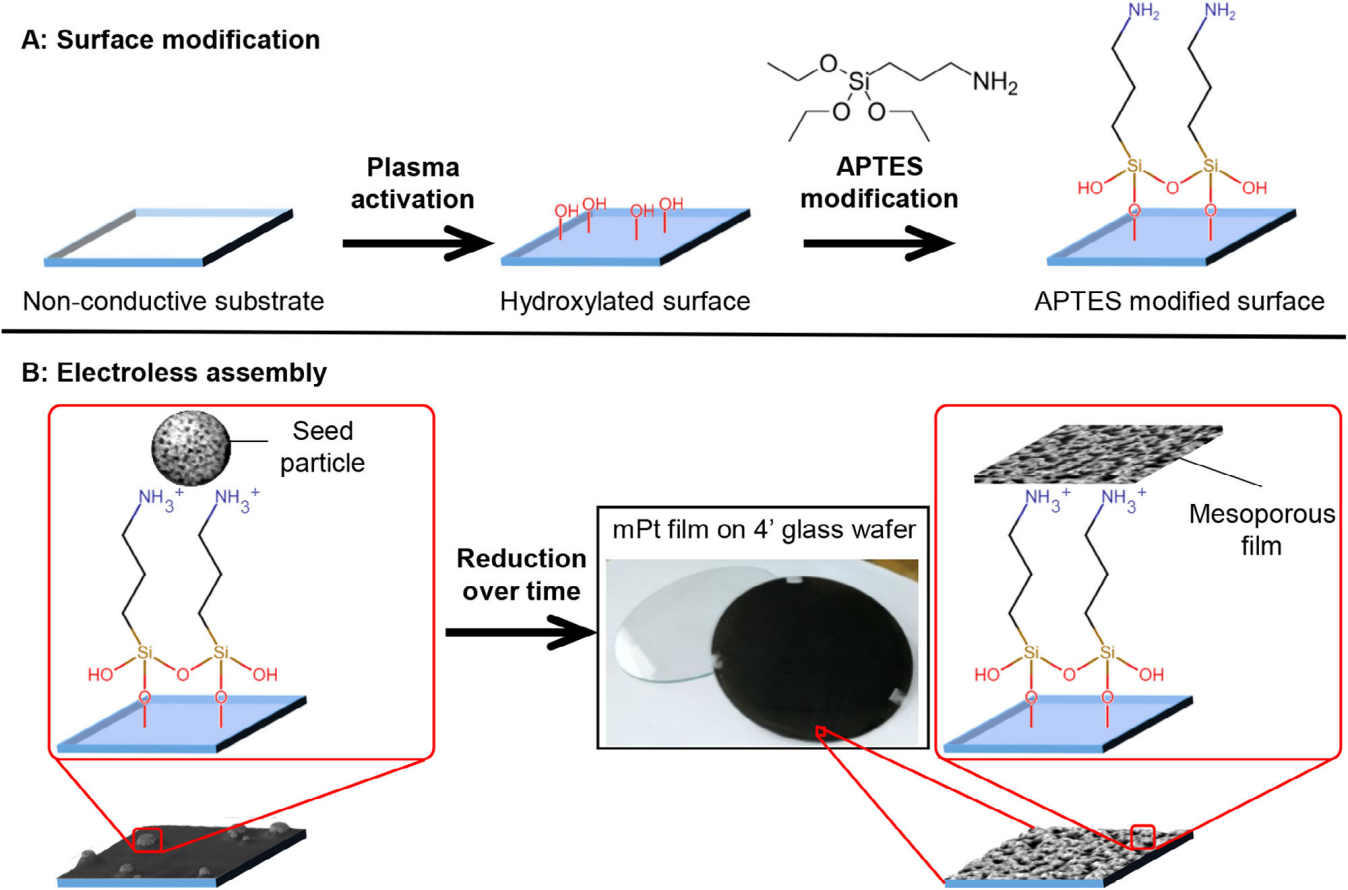
Outline of the electroless assembly of mesoporous metal film on non‐conductive substrate. A) Surface modification of APTES and B) electroless assembly of mesoporous metal film. The square marks observed on the mPt film deposited on the 4‐inch glass wafer are anchor points to secure wafer during electroless assembly.

Glass‐supported mPt film was synthesized via electroless micelle‐assisted deposition on an APTES‐functionalized glass substrate (**Figure** **1**). First, ozone/UV irradiation generates ─OH group, which forms a covalent bond with the Si─OH on hydrolyzed APTES molecule, at the glass surface.^[^ ^22^, ^24^ ^]^ The APTES molecules form a very stable and resistant monolayer on the glass by self‐polymerization.^[^ ^22^, ^23^, ^24^ ^]^ It is confirmed that the presence of these amine groups on the glass surface by high‐resolution X‐ray photoelectron spectroscopy (HR‐XPS). N 1*s* HR ‐ XPS spectra of glass substrate with and without APTES modification are shown in Figure S1a (Supporting Information). The peak ≈401 eV is attributed to the ─NH_3_^+^ which only appears in the APTES‐modified glass sample, which suggests that the amine group are protonated, and the APTES‐modified glass surface become positively charged. Also, there is an elevated concentration of N 1*s* on the APTES‐modified glass surfaces (8 at.%) compared to the glass without APTES modification (1 at.%). In the Fourier‐transform infrared (FT‐IR) spectrum, an additional shoulder near 1200 cm^−1^ is observed in the APTES‐modified glass sample but absent in the unmodified glass, which can be attributed to the C─N vibration of APTES molecules (Figure S1b, Supporting Information). Furthermore, the zeta potential analysis reveals that the potential of the APTES‐modified glass sample is +10.24 ± 1.28 mV (Figure S2, Supporting Information), compared to −19.38 ± 2.28 mV (Figure S3, Supporting Information) for the unmodified glass. The combined HR‐XPS, FT‐IR and zeta potential results strongly suggest that the modification of glass surface creates a positively charged surface due to the presence of amine groups derived from APTES molecules.

Unlike conventional electroless methods, our system employs ascorbic acid as a mild, non‐toxic reducing agent and Pluronic F127 (Poly(ethylene oxide)‐poly(propylene oxide)‐poly(ethylene oxide) block copolymer, PEO_100_‐b‐PPO_65_‐b‐PEO_100_) as a structure‐directing template to guide the formation of well‐defined mesopores within the growing Pt film. Traditional electroless deposition is rarely applied to Pt due to its high reduction potential and the absence of effective surface anchoring strategies. Our approach overcomes these limitations by synergistically combining surface chemical functionalization with micelle templating. In particular, APTES modification introduces protonated amine groups (─NH_3_^+^) on the substrate surface, which electrostatically attract [PtCl_6_]^2−^ anions from the precursor solution (Figure 1). This interaction promotes localized precursor concentration and site‐selective nucleation of Pt. Subsequent reduction of these ions by ascorbic acid occurs concurrently with micelle assembly, leading to the formation of continuous mPt films. Importantly, this electroless deposition process is independent of substrate conductivity, thereby enabling the fabrication of mPt films on a wide variety of non‐conductive substrates.

Further insights are provided by the results of monitoring the deposition of mPt onto the APTES‐modified glass surface over time. Figure S4 (Supporting Information) shows the photograph of the glass substrate that undergoes electroless assembly after 1, 2, and 20 h, and the color of the mPt samples become darker as the chemical reduction takes place. The SEM images show there are small‐sized particles, which are speculated to be Pt seed particles (Figure S4, Supporting Information, red circles), attached to the APTES‐modified glass surface. As the chemical reduction proceeds, these seed particles are then grown into mPt nano‐ and micro‐particles; and ultimately into a mPt film. Unlike the electrochemical method, the current electroless approach has no restriction on shape and size of glass substrate and we have successfully fabricated continuous mPt film on a 4‐inch glass wafer and on a circular Kapton tape with a diameter of 3 cm (Figure 1; Figure S5, Supporting Information). The synthesized mPt films have a black metallic reflective appearance which covers the whole area of the glass and Kapton substrates. The mPt film supported on Kapton tape, exhibits excellent flexibility (Figure S5, Supporting Information). This demonstrates its potential for integration into wearable devices.

**Figure 2:**
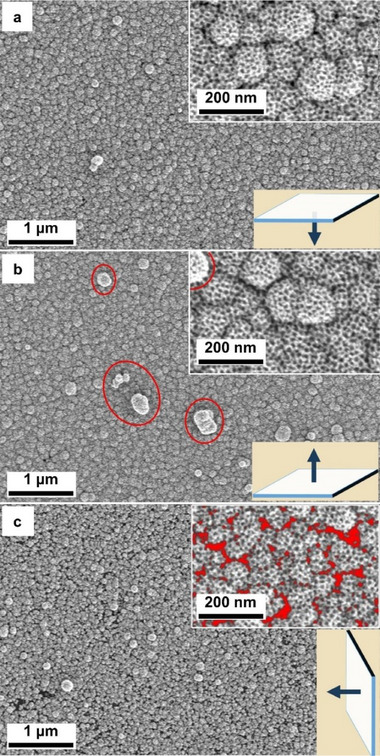
SEM images of mPt film synthesized at various orientations of the APTES‐modified glass surface: a) downwards, b) upwards, and c) at 90°, as illustrated in the insets at the bottom right, where arrows indicate the direction of the APTES‐modified surface. The red circles in b) highlight the depositing of large particles and the red areas in (c) highlight the gaps in non‐continuous film.

Furthermore, SEM results (**Figure** **2**; Figure S5, Supporting Information) show that mesoporous structures of the Pt films are successfully synthesized on glass and Kapton substrates by electroless assembly method. Depending on the orientation of the APTES‐modified glass surface in the reaction medium, the number of mesoporous particles attached onto the mPt film and its continuity varies. As shown in Figure 2a, the mPt films appear to have the best surface quality when the glass substrate is oriented with the APTES‐modified surface facing downward as compared to the upward and 90° orientation. There are unwanted large particles attached to the mPt film when the modified surface is facing upward (Figure 2b), and the film appears non‐continuous when the modified surface is oriented perpendicular in the solution (Figure 2c). As a result, all the mPt films synthesized after are in an orientation with the APTES‐modified surface facing downwards unless specified otherwise. The porous structure of the mPt films on non‐conductive substrates is similar to those in the mesoporous metal film synthesized by electrochemical deposition, which indicates that the F127 block copolymer micelles in the reaction mixture act as a soft‐template to create mesoporous structure in the same way as to the conventional electrochemical micelle assembly of mesoporous metal film.^[^ ^29^ ^]^ The F127 block copolymer micelles in the reaction mixture are believed to direct pore formation. To verify the essential role of micelle templating, a control experiment was conducted in the absence of F127 under identical conditions. As shown in Figure S6 (Supporting Information), no continuous mesoporous film is formed; instead, Pt aggregates are deposited on the APTES‐modified glass surface. This contrast clearly demonstrates that the presence of F127 micelles is crucial for forming the continuous mesoporous structures, providing direct evidence of their templating function in the electroless assembly process.

**Figure 3:**
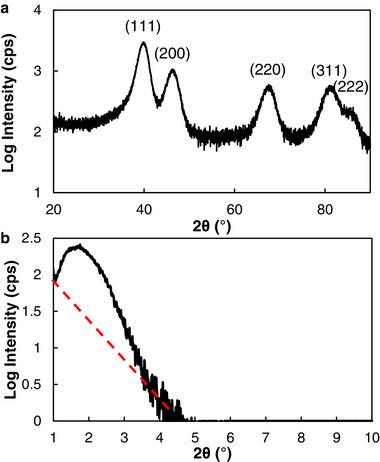
a) Wide‐angle and b) low‐angle XRD spectra of mesoporous Pt film synthesized by electroless assembly. The red dashed line is to highlight the peak around 2°.

The crystallographic structure of the mPt film deposited on a glass substrate was characterized using X‐ray diffraction (XRD). The wide‐angle XRD pattern (**Figure** **3**a) displays five distinct diffraction peaks located at 40, 47, 68, 81, and 86°, which correspond to the (111), (200), (220), (311), and (222) crystallographic planes, respectively. These reflections are characteristic of a face‐centered cubic crystal structure.^[^ ^30^, ^31^ ^]^ To investigate the porous structure of mPt film, low‐angle XRD analysis was performed. The low‐angle XRD pattern reveals a board diffraction peak centered at 1.75° (Figure 3b), which is indicative of a mesoporous architecture. This board peak ranges from 3 to 8 nm. The pore‐to‐pore distance observed by SEM (Figure S4, Supporting Information) falls within the range. The XRD data confirms that the mPt film exhibits a well‐defined mesoporous structure.

**Figure 4:**
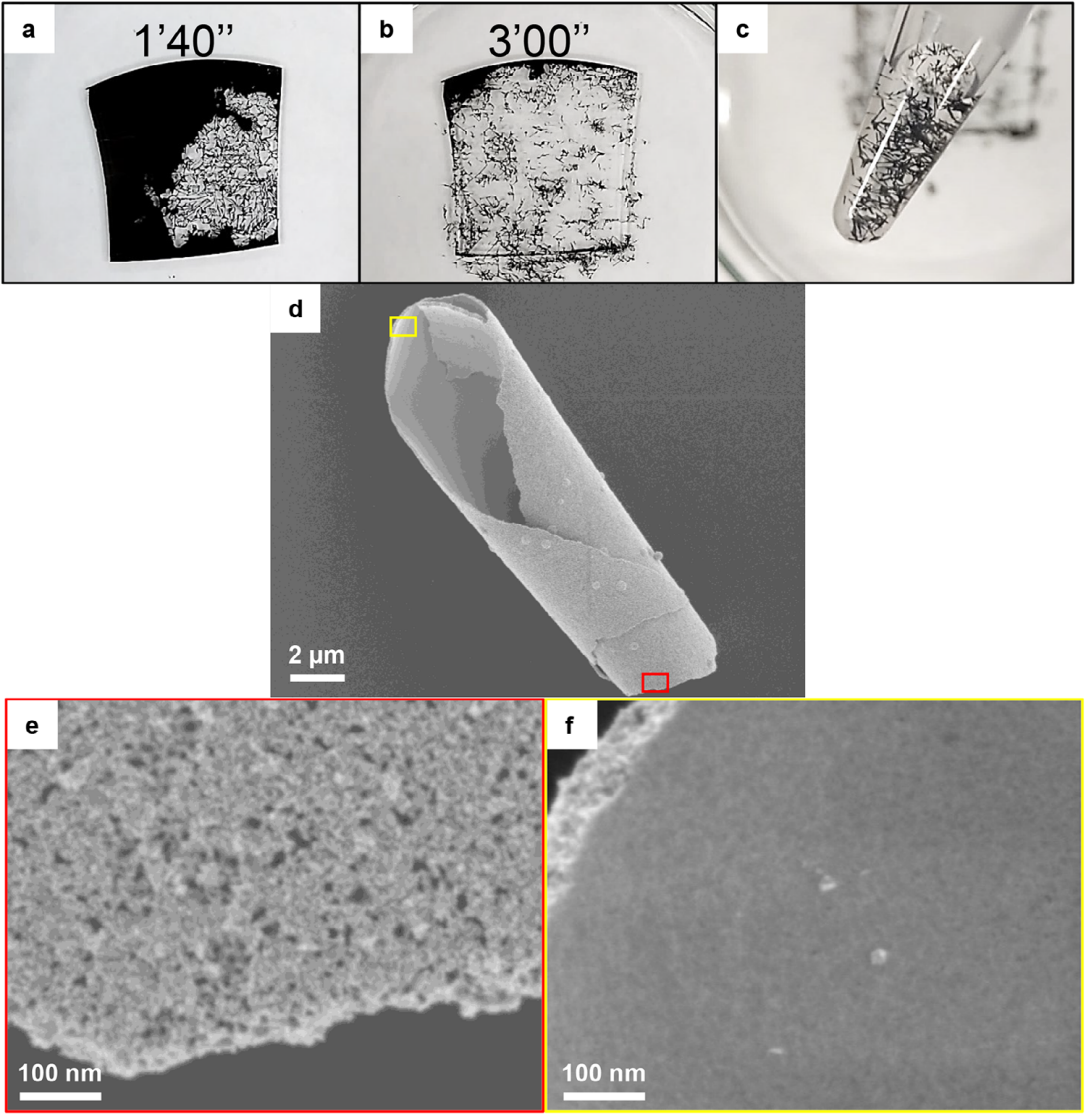
Photographs of mesoporous Pt film supported on 2 × 2 cm glass immerged in 8 m KOH a) after ≈2 min and b) 3 min, and c) the collected self‐standing film. d) is the SEM image of detached mPt film showing the rolled structure. e) and f) are the SEM images showing the outer and internal walls, respectively.

The glass‐supported mPt film exhibits strong adhesion, as evidenced by its resistance to mechanical removal via peel‐off methods using sticky tape (see Video S1 in the Supporting Information). The film remains firmly attached to the substrate after peeling, indicating mechanical stability under mild external stress. This strong attachment is attributed to the chemical interaction between the mPt film and the amino‐functionalized glass surface. However, this adhesion can be disrupted chemically using an alkaline solution. When the glass‐supported mPt film is treated with potassium hydroxide (KOH) solution, the film detaches from the substrate, and metallic black‐colored solids are released (**Figure** **4**; Video S2, Supporting Information). Representative snapshots showing the release process upon treatment with 8 m KOH solution are provided in Figure 4a–c. Complete lift‐off of the mPt film occurs within ≈3 min. The released solids appear as black needle‐shaped structures, which were subsequently examined by SEM. Notably, the morphology of the detached, self‐supporting mPt film remains highly porous (Figure 4d–f), indicating that the structural integrity of the mesoporous network is preserved after lift‐off. The rate of the lift‐off process is correlated with the concentration of KOH solution, as higher concentrations accelerate the release of mPt film. Nonetheless, no significant morphological differences are observed in the detached mPt films obtained using varying KOH concentrations, as confirmed by SEM analysis (Figure S7, Supporting Information). These results suggest that while the films are mechanically stable under mild external stress (e.g., peel‐off), their adhesion to the substrate can be disrupted through chemical treatment (e.g., KOH lift‐off). Nonetheless, a comprehensive evaluation of long‐term mechanical and environmental durability, including under cyclic stress and variable humidity and temperature conditions, will be necessary to fully validate their suitability for practical applications, particularly in flexible or wearable devices.

Intriguingly, the detachment of the mPt film from the glass substrate following KOH treatment, resulting in fragmented but structurally intact rolled‐up mPt segments (Figure 4d). Transmission electron microscopy (TEM) imaging (Figure S8, Supporting Information) confirms that the entire mPt film retains a porous architecture after KOH solution treatment. However, SEM analysis reveals a clear contrast between the outer and inner surfaces: the outer surface is characterized by well‐defined open pores, while the inner surface appears relatively smooth and non‐porous. This morphological asymmetry is likely a consequence of the deposition mechanism. During the initial stage of deposition, strong interactions between the nascent platinum seed particles and the surface‐bound ─NH_3_^+^ groups disrupt the organization of the micelle templates near the substrate interface. As a result, templated pore formation is suppressed in this region, leading to the formation of a dense, non‐porous layer on the side that was in contact with the glass. In contrast, the film which grows away from the glass surface retains the typical mesoporous structure directed by the micelle templates. This asymmetry in surface morphology likely contributes to the spontaneous rolling of the films upon detachment. The dense, non‐porous inner layer and the porous outer layer create a stress gradient across the film thickness. This differential mechanical stress causes the film to bend and roll into cylindrical shapes.

**Figure 5:**
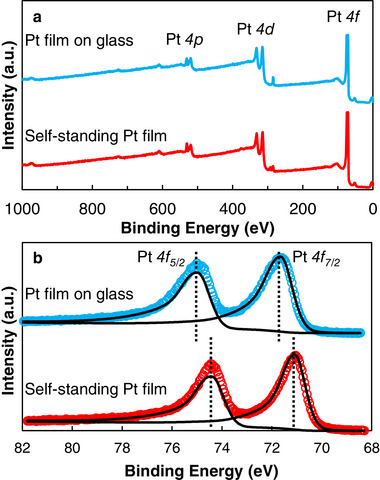
a) XPS survey spectra and b) Pt 4*f* XPS spectra of glass‐supported (blue) and self‐standing mPt film (red). The fitted spectra are shown as black curves and the dotted line indicating the location of 4*f*_7/2_ and 4*f*_5/2_ peaks.

The compositions and electronic states of glass‐supported and self‐standing mPt film were measured by XPS (**Figure** **5**). The XPS survey spectra of these mPt films are shown in Figure 5a indicating the presence of Pt. Figure 5b shows the Pt 4*f* spectra and the peaks centering at 75.0 and 71.7 eV for glass‐supported mPt film; and 74.4 and 71.1 eV for self‐standing mPt film, respectively. The 3.3 eV energy separation of the Pt 4*f* peaks agrees with the 4*f*_7/2_ and 4*f*_5/2_ level splitting due to spin‐orbit interaction. Pt 4*f* peaks suggest a mixture of metallic platinum and oxidized platinum species. For glass‐supported mPt film, the surface potential charge of the glass substrate shifted the photoelectron peaks to a higher binding energy due to the surface oxidization of film.^[^ ^32^ ^]^ As shown in Figure S9 (Supporting Information), the O 1*s* peak for glass‐supported mPt film was resolved into three 531.1, 532.1, and 533.5 eV subpeaks corresponding to O^2−^, O^−^, and oxygen species adsorbed on the mPt film surface, respectively. In contrast, the subpeak for surface oxygen species is absent after lift‐off. This difference in the O 1*s* peaks observed can be attributed to the KOH treatment during the lift‐off process, which may remove surface oxygen species from the mPt film.

**Figure 6:**
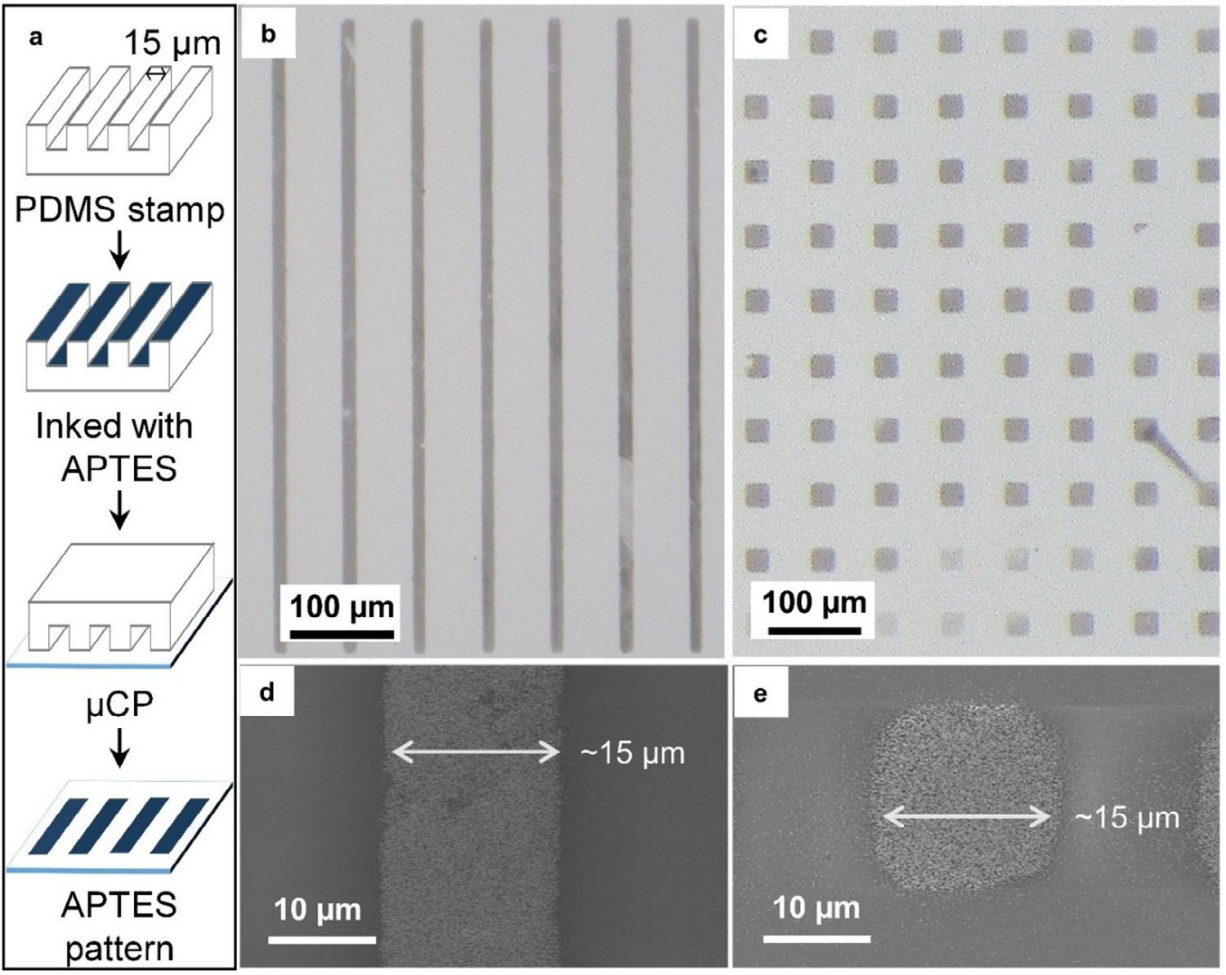
a) Outline of µCP of APTES patterns on glass surface. b) and c) are micrographs of the fabricated mesoporous Pt patterns on glass substrate. d) and e) are the corresponding SEM images of mesoporous Pt patterns.

As a proof‐of‐concept, mPt patterns on a glass substrate were fabricated using the well‐known µCP technique. This involves printing microarrays of APTES on the glass surface, followed by electroless micelle assembly. The details of the µCP process for APTES on the glass surface are illustrated in **Figure** **6**a. Briefly, a SU‐8 master mold containing micropatterns was fabricated using photolithography (Figure 6b). Polydimethylsiloxane (PDMS), a silicone polymer, was used to create stamps (Figure 6c) with an inverse copy of the pattern from the SU‐8 master mold. The PDMS stamps were inked with an APTES solution and then brought into contact with the plasma‐activated glass surface. As a result, APTES molecules only bond covalently to the glass in the contacted areas, forming the micropatterns on the surface.^[^ ^23^, ^25^ ^]^ These APTES micropatterns then undergo electrostatic interactions with deposited mPt during the electroless reduction reaction of the mPt film. The resulting mPt patterns are shown in Figure 6d,e, with the width of the line pattern measuring ≈15 µm, which matches the micropattern on the PDMS stamp. To the best of our knowledge, this is the first example of fabricating mPt micropatterns by µCP.

To verify whether the mPt film synthesized by electroless method exhibits chemical activity comparable to that of conventional platinum, mPt film was deposited on fluorine‐doped tin oxide (FTO) glass and tested as an electrochemical catalyst. It is important to note that the use of FTO in this context was primarily to facilitate electrochemical testing and to serve as proof‐of‐concept for the functional utility of the mPt film synthesized by electroless method as an electrode material. Although the chemicals used in the synthesis of mPt films are thoroughly removed using organic solvent, trace amounts remain strongly adsorbed on the surface and are difficult to eliminate completely, potentially affecting electrochemical performance. To remove these residual impurities, the mPt films were sintered in air at 350 °C for 12 h. SEM analysis confirms that the porous structure of the mPt film is successfully synthesized on FTO glass and remains intact after sintering (Figure S10a,b, Supporting Information). The mPt film exhibits intimate contact with the FTO substrate, enabling efficient electron transfer, and its thickness is ≈820 nm, as observed in the cross‐sectional SEM image (Figure S10c, Supporting Information).

**Figure 7:**
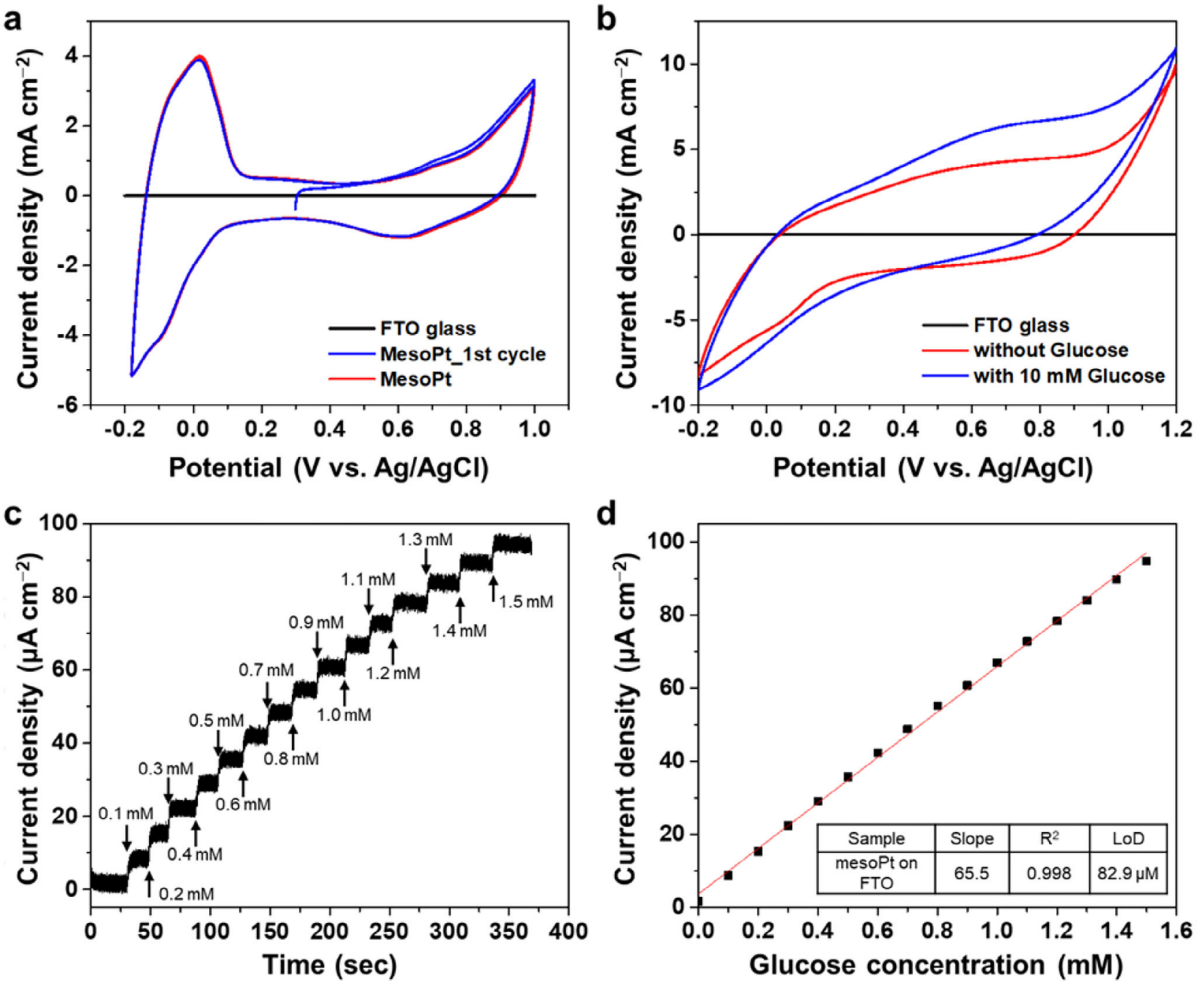
a) CV curves of bare FTO glass and mPt film on FTO glass measured in 0.5 m H_2_SO_4_ solution within the potential range of −0.18  to 1.0 V (vs Ag/AgCl) at a scan rate of 20 mV s^−1^. b) CV curves of mPt on FTO glass measured in 0.1 m PBS solution before (red) and after the addition of 10 mm glucose (blue), measured within the potential range of −0.2 to 1.2 V at a scan rate of 100 mV s^−1^. c) Amperometric response of mPt film on FTO glass to stepwise additions of glucose into 0.1 m PBS solution at a constant potential of 0.6 V. d) Calibration curve showing the current response as a function of glucose concentration, demonstrating glucose sensing performance of mPt film. A Pt wire and an Ag/AgCl (saturated KCl) electrode were used as the counter and reference electrodes, respectively. The currents are normalized by geometrical substrate area.

To evaluate the effect of the pore structure in mPt film and its electrochemical activity, the electrochemically active surface area (ECSA) of mPt film deposited on FTO glass (Figure S10, Supporting Information) was measured via cyclic voltammetry (CV) (**Figure** **7**a). A three‐electrode configuration was employed, comprising mPt film on FTO glass as the working electrode, a platinum wire as the counter electrode, and an Ag/AgCl electrode as the reference electrode. Hydrogen underpotential deposition was measured by scanning the potential from −0.18  to 1.0 V at a rate of 20 mV s^−1^ in 0.5 m H_2_SO_4_. Unlike the FTO glass, which exhibits negligible electrochemical activity in this range, the heat‐treated mPt film displays two pairs of redox peaks between −0.18 and 0.1 V, corresponding to the adsorption and desorption of hydrogen atoms on Pt.^[^ ^33^, ^34^ ^]^ The overlapping of these peaks likely reflects the polycrystalline nature of the mPt film (Figure 3a). From the hydrogen desorption peak, the ECSA is calculated to be 60.8 cm_Pt_^2^. The as‐prepared mPt film exhibits structural degradation under repeated electrochemical cycling (Figure S11, Supporting Information), indicating electrochemical instability. Consequently, the electrochemical properties of the mPt films were further investigated using heat‐treated samples. To compare our material with other mesoporous metal films, we calculated the volume‐normalized ECSA using a geometrical film area of 0.46 cm^2^ and a measured thickness of 820 nm. The resulting value of 161.1 m^2^ cm^−3^ surpasses those reported for most previously published mesoporous metal films (Table S1, Supporting Information), underscoring the effectiveness of our synthesis approach in exposing internal surface area—an essential advantage for electrochemical applications. Leveraging this large ECSA, the heat‐treated mPt film was applied to electrochemical glucose sensing.

Given the rising global incidence of diabetes and other glucose‐related metabolic disorders, there is an increasing demand for accurate, sensitive, and reliable glucose sensing technologies.^[^ ^35^ ^]^ In this study, non‐enzymatic glucose sensing was selected as a representative application to assess the electrochemical performance of the mPt films. The CV measurements (Figure 7b) were conducted in 0.1 m phosphate buffer (PBS, pH 7.4) to mimic physiological conditions and ensure the relevance of the results to potential biomedical applications. CV was employed as the initial method to determine the glucose oxidation behavior on the mPt film. Upon the addition of 10 mm glucose to the PBS, the CV profile of the mPt electrode (Figure 7b, blue curve) shows a marked increase in anodic current over the potential range of 0.1 to 1.2 V, along with the appearance of a new oxidation peak at 0.6 V.

Amperometric measurements were conducted to evaluate the electrochemical response of the mPt electrode to successive additions of glucose, with the applied potential held at 0.6 V versus Ag/AgCl (Figure 7c). Upon each addition of glucose, a sharp increase in current is observed, indicating a rapid onset of the glucose oxidation reaction. This response stabilizes quickly, forming a plateau within seconds, suggesting that the system reaches a steady‐state condition promptly after each injection. However, a minor decrease in the current is noted over successive additions, despite continuous magnetic stirring of the electrolyte. This decline can be attributed to two factors: (1) diffusion limitations within the deep, tortuous mesoporous structure of the mPt film, which may hinder uniform glucose penetration and product removal, and (2) partial surface fouling caused by the adsorption of gluconolactone, the oxidation product of glucose, which can block active sites and reduce catalytic efficiency over time.

The calibration plot (Figure 7d) demonstrates a linear correlation between glucose concentration and oxidation current, confirming the quantitative response of the mPt sensor within the tested concentration range. The sensitivity of the mPt‐based glucose sensor is calculated to be 65.5 µA cm^−2^ mm ^−1^, reflecting the effective electrocatalytic activity and high ECSA of the mesoporous structure. Despite this, the limit of detection (LoD) is determined to be 82.9 µm, which is relatively high when compared to state‐of‐the‐art non‐enzymatic glucose sensors. This limitation is attributed to a considerable level of background noise, likely originating from capacitive currents of the electrode and diffusion constraints imposed by the molecular size of glucose.

## 3 Conclusion

We have demonstrated the electroless deposition of mPt on non‐conductive substrates. By leveraging the interaction between APTES‐modified surfaces and metal precursors, while utilizing polymer micelles as a soft template, we have successfully synthesized mPt films on both rigid glass and flexible substrates such as Kapton tape. This electroless approach overcomes the limitations of traditional electrochemical methods, which are restricted to conductive substrates. We have also achieved detachment of the mPt film from the glass substrate following KOH treatment, resulting in fragmented but structurally intact rolled‐up mPt segments observable under SEM. Furthermore, the integration of µCP technique with this electroless method enables the fabrication of mPt micropatterns with precise control over their dimensions. The mPt film fabricated on FTO glass exhibits a sensitivity of 65.5 µA cm^−2^ mm ^−1^ and a LoD of 82.9 µM in non‐enzymatic glucose sensing, demonstrating the potential of mPt for electrochemical applications. This work presents a simple and effective approach to fabricating patterned mesoporous materials, which opens new avenues for their utilization in a wider range of applications beyond conventional electrochemical sensing and electrocatalysis, particularly those requiring integration with non‐conductive materials.

## 4 Experimental Section

### Reagents and Materials

Hydrogen Tetrachloroaurate(III) Trihydrate (HPt·3H_2_O, Fuji Film Wako Ind. Co., ACS Reagent Grade), 3‐aminopropyltriethoxysilane (APTES, Tokyo Chemical Industry Co., Ltd., >98%), L‐ascorbic acid (Sigma‐Aldrich, 99%), Pluronic F‐127 (Sigma‐Aldrich, BioReagent), SU‐8 3050 (MicroChem Corp.), and poly‐(dimethylsiloxane) (PDMS, Silpot 184, Dow Corning Toray Co., Ltd.), 2‐methoxy‐1‐methylethyl acetate (Fuji Film Wako Ind. Co., 97%), ethanol (Fuji Film Wako Ind. Co., 99.5%), acetone (Fuji Film Wako Ind. Co., 99%), and potassium hydroxide solution (Fuji Film Wako Ind. Co., 1 and 8 molL^−1^) were used as received.

### Electroless Micelle Assembly of mPt Film on Glass

For a typical electroless micelle assembly of mPt film (Figure 1), a glass substrate is treated with Ozone for 7 min, followed by 5 min UV exposure to clean and generate OH group on glass surface. This surface activated glass substrate was then rinsed with milliQ water thoroughly before submerging in a 10% APTES aqueous solution for 1 h to covalently bond APTES onto the glass surface. This APTES treated glass was then immersed into a reaction solution for the electroless micelle assembly of mPt film containing 150 mg of F127, 10 mL of 20 mm HPt and heated to 50 °C. Then 10 mL of 100 mm ascorbic acid was added into the solution mixture to start the reduction reaction to allow formation of the mPt film overnight. This combination of precursors and conditions is deliberately chosen to enable the formation of mesoporous Pt under relatively mild and environmentally benign conditions. The film supported on the glass substrate was removed from the reaction mixture and ultrasound cleaned in ethanol and then in water before further analysis. The film attached on glass substrate was submerge in an 1 or 8 mol L^−1^ KOH solution to release the mPt film from glass substrate and the self‐standing films were collected as metallic black‐colored solids.

### Microcontact Printing of APTES on Glass Substrate

A SU‐8 master mold for PDMS stamp were fabricated using soft lithography.^[^ ^25^ ^]^ In brief, micropattern designs comprised of 15 µm wide stripes and 15 µm × 15 µm square were designed with AutoCAD (AutoDesk, USA). A layer of 20 µm negative epoxy photoresist, SU‐8 3050, was coated onto a 4‐inch silicon wafer with a spin coater (1H‐DX2, Mikasa Co., Ltd., Japan). The features of PDMS stamp were patterned by photolithography using a mask aligner (PEM‐800, Union Optical Co., Ltd., Japan) with a 200 mJ cm^−2^ UV exposure. The SU‐8 patterns were developed using 2‐methoxy‐1‐methylethyl acetate. PDMS was premixed with a curing agent at a ratio of 10:1, followed by stirring and degassing under reduced pressure for 3 min with a planetary centrifugal mixer (Awatori‐rentaro ARV‐310, THINKY, Ltd., Japan) before being poured onto the SU‐8 master mold. Stamps with the inverse copy of the pattern present on the SU‐8 master mold were obtained, after desiccated and cured for 45 min at 80 °C on a hot plate. The PDMS stamps were inked with 10% by volume of APTES aqueous solution for 15 min and then rinsed with Milli‐Q water (Millipore, Japan) which followed by drying under N_2_ gas. The inked PDMS stamp was then brought into contact with an Ozone/UV activated glass substrate for 10 sec.

### Imaging and Characterization

The surface morphology of the mPt films supported on various substrates were imaged directly using the Zeiss GeminiSEM 560 instrument at 2 kV. For the self‐standing mPt film, a diluted ethanol dispersion was prepared and deposited onto the surface of a copper microgrid with supported carbon membrane. The sample was then air‐dried before being affixed to a STEM holder for SEM and TEM observation. XRD analyses were performed using Rigaku SmartLab diffractometers (Rigaku, Japan) equipped with CuKα radiation, with scan rates set at 2° min^−1^ employing the parafocusing optics. HR ‐ XPS measurements were conducted using PHI Quantes (ULVAC‐PHI, Inc.). Zeta potential and FT‐IR spectra of glass substrates with and without APTES modification were measured using a Zeta‐potential and particle size Analyzer (ELSZ‐2000ZS, Otsuka Electronics Co., Ltd.) and a JASCO FT/IR‐4X spectrometer with an attenuated total reflection attachment, respectively. CV curves of FTO glass and mesoporous Pt film on FTO glass were measured in 0.5 m H_2_SO_4_ solution with the potential window of −0.18 to 1.0 V at a scan rate of 20 mV s^−1^. Glucose detection of mesoporous Pt film on FTO glass was performed at a constant potential of 0.6 V in 0.1 m phosphate‐buffered saline (pH 7.4) and 10 mm glucose was added stepwise. A Pt wire and an Ag/AgCl (saturated KCl) electrode were used as the counter and reference electrodes, respectively. The ECSA was calculated using the charge associated with hydrogen desorption by integration as follows (this charge is directly proportional to the active surface area of mPt):

(1) $$\begin{array}{ccc} \mathrm{Charge}\;\left(Q\right) & & =\mathrm{Integration}\mathrm{of}\mathrm{peaks}\mathrm{corresponding}\mathrm{to}H\mathrm{desorption}\;\left(mAV\right)/\\ & & scan\;\mathrm{rate}\;\left(Vs^{-1}\right) \end{array}$$

(2) $$\mathrm{ECSA}=Q\;\left(mC\right)/Q_{\mathrm{ref}}\left(\mu Ccm^{-2}\right)$$

where Q_ref_ = 210 µC cm^−2^ is the charge of a hydrogen monolayer adsorbed on polycrystalline platinum.

## Conflict of Interest

The authors declare no conflict of interest.

## Supporting information



Supporting Information

Supplemental Video 1

Supplemental Video 2

## Data Availability

The data that support the findings of this study are available from the corresponding author upon reasonable request.
