# Supplementary material for: Soft‐Templated Electroless Synthesis of Mesoporous Metal Films on Non‐Conductive Substrates
Source: Small. 2025 Aug 19;21(38):2505676. doi: 10.1002/smll.202505676 (PMC12462553; doi:10.1002/smll.202505676)
Supplement: Supplementary file 1 — Supporting Information [file SMLL-21-2505676-s003.docx]

Supporting Information

Soft-templated Electroless Synthesis of Mesoporous Metal Films on Non-conductive Substrates

Mandy H. M. Leung, Hirokatsu Miyata, Tokihiko Yokoshima, Yusuke Asakura, Minsu Han, Daigo Natsuhara, Miharu Eguchi, Chia-Hung Liu, and Yusuke Yamauchi*

**Figure S1.** a) High-resolution XPS spectra of N 1*s* and b) FT-IR spectra of glass substrate with (red) and without (blue) APTES modification. The fittings are shown as black dashed curves, and the calculated atomic concentration is about 8 % for the glass surfaces with APTES modification.

**Figure S2.** Zeta potential results of 3 independent measurements on APTES-modified glass.

**Figure S3.** Zeta potential results of 3 independent measurements on unmodified glass.

**Figure S4.** Top left is a photograph of the glass-supported mPt film synthesized after 20 h, 2 h and 1 h (from top to bottom). SEM image of glass substrate after 1 h, 2 h and 20 h of electroless micelle assembly chemical reduction reaction. The red circles highlighting the small-sized mPt seeds and the inset for 1 h showing the porous structure of the mPt nanoparticles attached to the glass. The pore-to-pore distance of mPt film is shown in the inset of 20 h.

**Figure S5.** mPt film supported on the non-adhesive surface of Kapton tape and the corresponding SEM image showing the mesoporous structure.

**Figure S6.** SEM image of APTES-modified glass substrate after 20 h of electroless chemical reduction reaction in the absence of F127.

**Figure S7.** SEM images of detached mPt films using 1 M KOH (top) and 8 M KOH solution (bottom).

**Figure S8.** TEM images of self-standing mPt film. The red box indicates the location of the bottom image.

**Figure S9.** O 1*s* XPS spectra of glass-supported (blue) and self-standing mPt film (red). The fitted spectra are shown as black curves.

**Figure S10.** SEM images of the mPt film on FTO glass: a) as-prepared, b) heat-treated at 350 °C for 12 h, and c) cross-sectional view.

**Figure S11.** CV curves of as-prepared mPt film on FTO glass measured in 0.5 M H_2_SO_4_ solution within the potential range of −0.2 to 0.6 V at a scan rate of 100 mV s^−1^.

| **Table S1.** Comparison of the ECSA of mesoporous metal films | | | | |
| --- | --- | --- | --- | --- |
| **Metal** | **Synthesis method** | **Volume-normalized ECSA**  **(m^2^/cm^3^)** | **Application** | **Reference** |
| Pt | Chemical | 161.1 | Glucose sensing | This work |
| Pt | Electrochemical | ~40 | Methanol oxidation | [1] |
| PtPdRhRuCu | Electrochemical | ~90 | Methanol oxidation | [2] |
| Pd | Electrochemical | ~140 | Ethanol oxidation | [3] |
| Pd | Electrochemical | ~220 | Ethanol oxidation | [4] |
| Au | Electrochemical | ~50 | SERS sensing | [5] |

[1] H. Wang, L. Wang, T. Sato, Y. Sakamoto, S. Tominaka, K. Miyasaka, N. Miyamoto, Y. Nemoto, O. Terasaki, Y. Yamauchi, *Chem. Mater.* **2012**, *24*, 1591.

[2] L. Fu, H. N. Nam, J. Zhou, Y. Kang, K. Wang, Z. Zhou, Y. Zhao, L. Zhu, R. Nandan, M. Eguchi, Q. M. Phung, T. Yokoshima, K. Wu, Y. Yamauchi, *ACS Nano*, **2024**, *18*, 27617.

[3] M. Iqbal, Y.V. Kaneti, K. Kashimura, M. Yoshino, B. Jiang, C. Li, B. Yuliarto, Y. Bando, Y. Sugahara, Y. Yamauchi, *Nanoscale Horizons* **2019**, *4*, 960.

[4] M. Iqbal, C. Li, K. Wood, B. Jiang, T. Takei, O. Dag, D. Baba, A.S. Nugraha, T. Asahi, A.E. Whitten, M.S.A. Hossain, *Chem. Mater.* **2017**, *29*, 6405.

[5] C. Li, Ö. Dag, T. D. Dao, T. Nagao, Y. Sakamoto, T. Kimura, O. Terasaki, Y. Yamauchi, *Nat. Commun.*, **2015**, *6*, 6608.
